# Intermittent Echocardiographic Monitoring During Superior Vena Cava Recanalization: A Protocol for Early Recognition and Management of Hemopericardium

**DOI:** 10.1186/s42155-025-00524-3

**Published:** 2025-02-20

**Authors:** Kara M. Fitzgerald, David S. Shin, Eric J. Monroe, Matthew Abad Santos, Ethan Hua, Jeffrey Forris Beecham Chick

**Affiliations:** 1 Section of Vascular and Interventional Radiology, Department of Radiology, University of Washington, Seattle, WA USA; 2 Division of Vascular and Interventional Radiology, Department of Radiology, University of Southern California, Los Angeles, CA USA; 3 Section of Interventional Radiology, Department of Radiology, University of Wisconsin, Madison, WI USA

**Keywords:** Cardiac tamponade, TCVO, SVC syndrome, Venous recanalization, Venous reconstruction

## Abstract

**Purpose:**

Endovascular recanalization and stent reconstruction constitute an effective and safe treatment option for symptomatic thoracic central venous obstruction (TCVO). Rare life-threatening adverse events are possible during or immediately following the procedure, with the most feared one being hemopericardium with cardiac tamponade. A technique to improve efficiency in detection and treatment of cardiac tamponade is described.

**Materials and methods:**

An institutional protocol was established for intraprocedural transthoracic echocardiographic monitoring during the TCVO recanalization procedures. The lower chest and upper abdomen were prepared within the sterile field. A separate stand was set up with unopened supplies needed for pericardial drain placement. Intermittent echocardiographic monitoring was performed throughout the TCVO procedure using a dedicated curvilinear probe prepared on the field. If indicated, an image guided pericardial drain was placed expeditiously.

**Results:**

Four cases of cardiac tamponade were encountered during or immediately post-procedure. All cases demonstrated technically successful and prompt pericardial drain placement with immediate reversal of the tamponade physiology.

**Conclusion:**

Echocardiographic monitoring during TCVO reconstruction by interventional radiologists is a useful technique which may aid in early diagnosis and management of cardiac tamponade.

## Introduction

Endovascular recanalization with stent reconstruction is a well-established treatment option for symptomatic thoracic central vein obstruction (TCVO). While rare, severe adverse events do occur [1–3]. The most frequent cause of procedure-related mortality is cardiac tamponade secondary to superior vena cava (SVC) injury resulting in hemopericardium [1]. Interventional radiologists should beware of cardiac tamponade and have a plan for expedited management when this life-threatening condition is suspected. This report describes a technique to improve efficiency in detection and treatment of cardiac tamponade. *Institutional review board approval was obtained for preparation of this report.*

## Materials & methods

### Technique

**Fig. 1:**
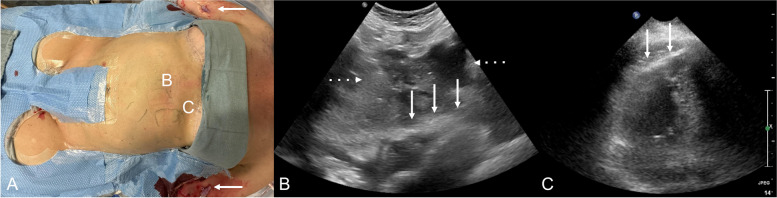
(A) Example of patient preparation for TCVO recanalization cases; white arrow indicating bilateral upper extremity access sites. “B” indicates subxiphoid view location and “C” indicates parasternal view location corresponding with images B and C location. (B) Subxiphoid echocardiogram view with white arrows indicating pericardial space with no effusion. Liver with multifocal metastasis (dashed arrows). (C) Parasternal echocardiogram view with arrows indicating pericardium. If pericardial drain was indicated, a parasternal approach would be preferred to avoid hepatic transgression

Technique is shown in Fig. 1. For complex TCVO recanalization and reconstruction (typically grade 3 or 4), the patient is placed under general anesthesia with continuous vital sign monitoring. A wide sterile preparation is performed including the bilateral arms, neck, chest, upper abdomen, and groin. A separate stand is prepared with materials needed for pericardial drain placement, including an access needle, a guide wire, dilators, and a 8- or 10-French (F) pigtail drainage catheter. A dedicated curvilinear (or curved array) ultrasound probe is prepared, and pre-intervention echocardiogram is performed to evaluate the baseline appearance of pericardial space and to identify a percutaneous window for potential drain placement (e.g., subxiphoid or parasternal view). The probe remains accessible throughout the case and is used to evaluate the pericardium intermittently throughout the procedure or immediately upon any hemodynamic compromise. If new pericardial effusion is noted with concurrent hemodynamic compromise, then pericardial drain placement is initiated using the Seldinger technique under ultrasound and fluoroscopic guidance. Venography is also performed for consideration of balloon tamponade or stent-graft placement. The patient is admitted post procedure, and the drain is removed after the patient has stabilized if the drain output remains minimal.

### Patient 1 (Fig. 2)

**Fig. 2:**
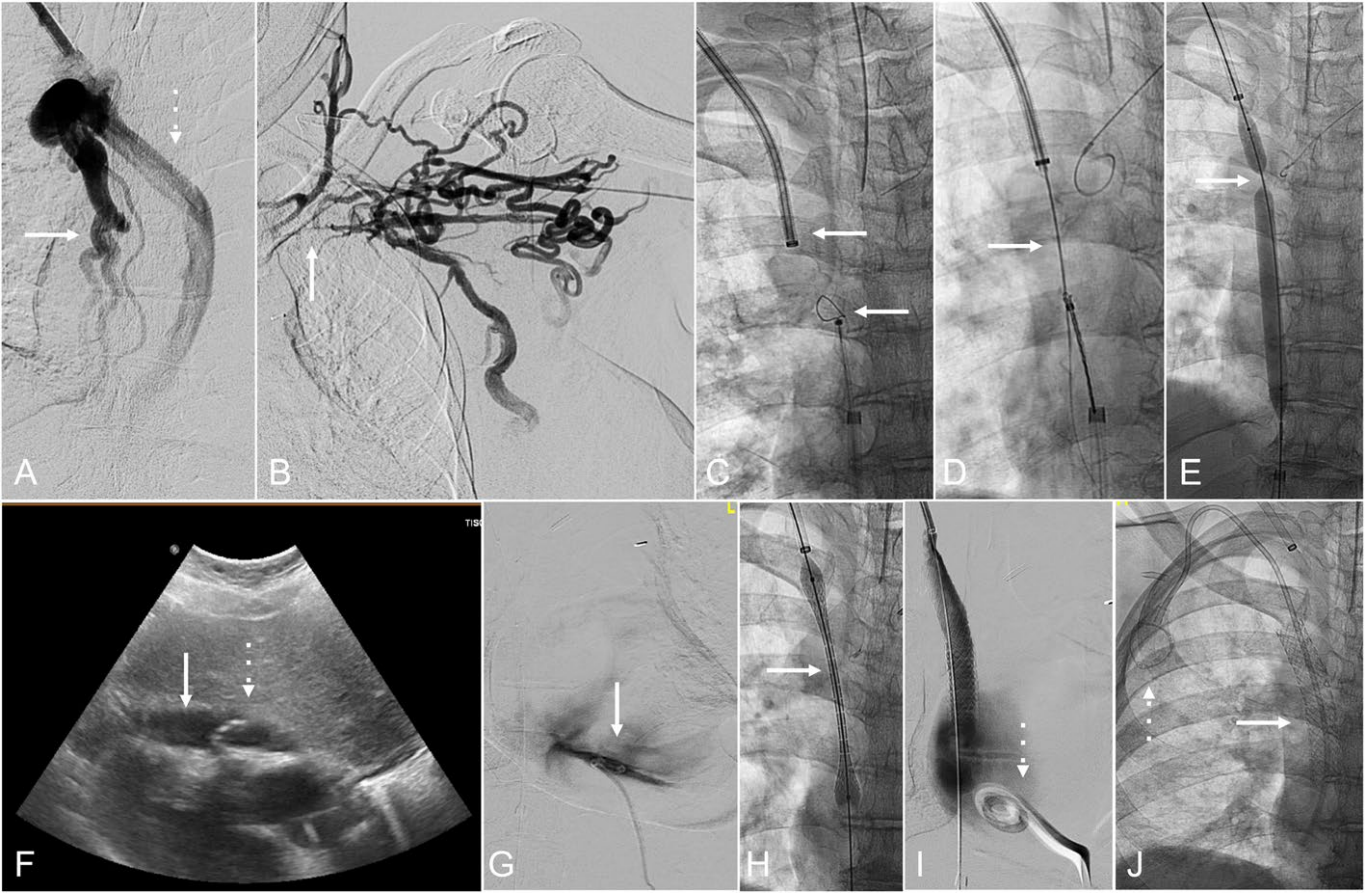
A 43-year-old female with sickle cell disease, with multiple prior central venous catheters, presented with acute bilateral breast swelling. (A) Venography from right IJV demonstrated occlusion of the superior vena cava (white arrow) with patency of the azygos vein (dashed white arrow). (B) Venography from left brachial vein demonstrated occlusion of the left subclavian vein (white arrow). (C) From the right IJV an 8-F sheath was placed (white arrow) and 10-F sheath and trilobe endovascular snare from right common femoral vein access to the right atrium/central superior vena cava (dashed white arrow). (D) From the right IJV, a 21-gauge Chiba needle (Cook Medical; Bloomington, IN) was advanced from sheath through the chronic SVC occlusion targeting the snare (dashed arrow) in superior vena cava/right atrium. (E) Balloon angioplasty after blunt recanalization with waist of balloon (arrow) at location of occlusion. (F) Subxiphoid US with pericardial effusion (arrow) and pericardial drain (dashed arrow). (G) Contrast injection confirming pericardial drain location. (H) Right brachiocephalocaval reconstruction with 11-mm VBX stent-graft (W. L. Gore & Associates; Flagstaff, AZ). (I) Central venography after stent-graft placement (arrow) with contrast filling the right atrium and no contrast extravasation. Pericardial drain (dashed arrow) in place. (J) Right IJV port catheter placed (dashed arrow) with catheter in brachiocephalocaval stent-graft

A 43-year-old female with sickle cell disease, with history of multiple indwelling central venous catheters, complicated by benign TCVO (Type 4 TCVO) with acute SVC syndrome (e.g., acute, painful bilateral chest swelling), presented with the need for central venous access. Baseline echocardiogram demonstrated no pericardial effusion. Right internal jugular vein (IJV), the left brachial vein, and the right common femoral vein (CFV) were accessed. Central venography was performed. Blunt recanalization was attempted but unsuccessful. Sharp recanalization was performed from the right internal jugular vein with a 21-gauge Chiba needle (Cook Medical; Bloomington, IN), crossing the chronic SVC occlusion and targeting a snare in the lower SVC. Angioplasty of the right brachiocephalic vein and SVC was performed. Acute hypotension was then noted and echocardiogram performed by the IR operator demonstrated a new moderate pericardial effusion. A venogram was immediately performed, and no extravasation was seen. Under ultrasound (US) and fluoroscopic guidance, a 12-F drain was placed into the pericardial space. Following rapid aspiration of approximately 300 mL of blood, the hemodynamics stabilized. Right brachiocephalocaval reconstruction was performed with a 11-mm VBX balloon expandable stent-graft (W. L. Gore & Associates; Flagstaff, AZ). The pericardial drain was removed on post procedure day (PPD) 4. The patient had symptoms of pericarditis and was treated with oral colchicine, 0.6 mg once daily for 90 days.

### Patient 2 (Fig. 3)

**Fig. 3:**
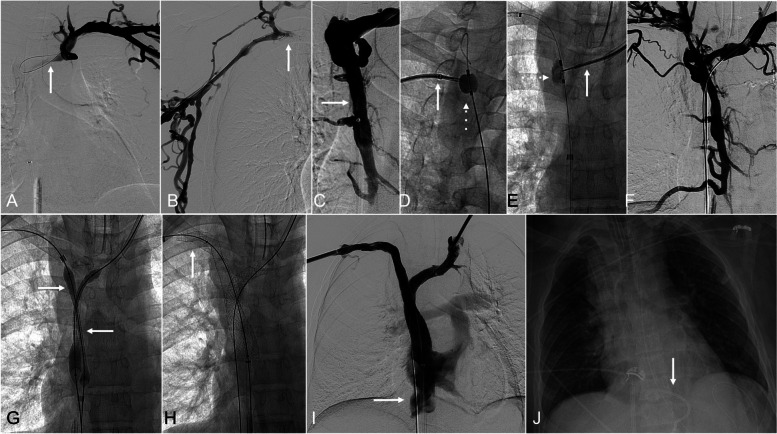
A 40-year-old male with end-stage renal disease on hemodialysis via left CFV tunneled dialysis catheter with bilateral upper extremity and chest venous engorgement and pain. (A & B) Bilateral upper extremity venography with left (A) and right (B) subclavian vein obstruction (arrows). (C) Retrograde opacification of the azygos vein (arrow) and collaterals. (D) Contrast filled compliant balloon (dashed arrow) directed from right lower in the SVC and Rösch-Uchida needle (Cook Medical; Bloomington, IN; arrow) directed from right upper extremity with sharp recanalization. (E) Rösch-Uchida needle (arrow) directed from left upper extremity after sharp recanalization of the left brachiocephalic obstruction targeting a compliant balloon in the SVC (dashed arrow) which is partially deflated. (F) Through-and-through wire access from bilateral upper extremity access to IVC with persistent filling of the azygos vein. (G) Deploying bilateral brachiocephalocaval 11-mm VBX stent-grafts (W. L. Gore & Associates; Flagstaff, AZ) directed from bilateral upper extremity access with balloon wasting noted (arrow). (H) Deployment of 10-mm Viabahn stent-graft (W. L. Gore & Associates; Flagstaff, AZ; arrow) to extend the stent construct peripherally in the right brachiocephalic vein. (I) Central venography after stent-graft deployment with in-line flow with reflux to the IVC (arrow). (J) After placement of pericardial drain (arrow)

A 40-year-old male with end-stage renal disease on hemodialysis via a tunneled left CFV catheter due to left brachiocephalic arteriovenous fistula thrombosis, presented with acute-on-chronic SVC syndrome (e.g., bilateral upper extremity and chest venous engorgement and pain) in the setting of Type 4 TCVO. Prior attempts at blunt recanalization of the TCVO were unsuccessful. Baseline echocardiogram demonstrated no pericardial effusion. Sharp recanalization was performed with a Rösch-Uchida needle set (Cook Medical; Bloomington, IN) advanced from the right brachial vein targeting a balloon in the SVC which was directed from the right CFV. A similar technique was used to recanalize the left BCV, with the Rösch-Uchida needle set directed from the left IJV and trilobe endovascular snare directed from the right CFV. Angioplasty of the SVC was performed in kissing fashion. Bilateral brachiocephalocaval 11-mm VBX stent-grafts were deployed in kissing fashion. Each brachiocephalic limb was extended peripherally with 10-mm Viabahn stent-grafts (W. L. Gore & Associates; Flagstaff, AZ). Post deployment balloon dilation was performed in kissing fashion. Hemodynamic change was then noted with hypoxia and hypotension. A venogram was performed and no extravasation was seen. Immediate echocardiogram revealed new pericardial fluid. An 8.5-F pericardial drain was placed with immediate return of 350 ml of blood and return of hemodynamic stability. The patient was admitted to the hospital. The pericardial drain was removed on PPD 4.

### Patient 3 (Fig. 4)

**Fig. 4:**
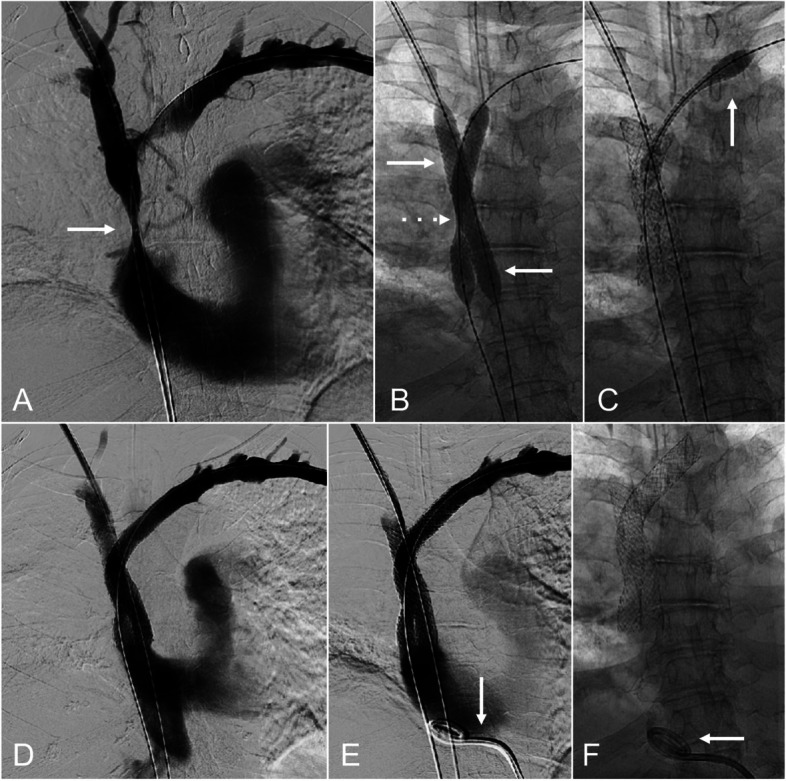
A 69-year-old female with non-small cell lung cancer with mediastinal mass and external compression of the SVC with acute on chronic bilateral upper extremity and facial swelling. (A) Central venography with through-and-through access from bilateral upper extremities to the IVC with moderate to severe stenosis of the SVC (arrow). (B) Deployment of bilateral brachiocephalocaval 11-mm VBX stent-grafts (W. L. Gore & Associates; Flagstaff, AZ), with waist (dashed arrow) on the left stent-graft and a well expanded right stent-graft. (C) Deployment of left brachiocephalic to subclavian 10-mm Viabahn stent-graft (W. L. Gore & Associates; Flagstaff, AZ; arrow) to extend the stent construct to cover the stenotic segments. (D) Central venography after stent-graft deployment with inline flow with reflux to the IVC. (E) Central venography after placement of pericardial drain (arrow). (F) After placement of pericardial drain (arrow)

A 69-year-old female with non-small cell lung cancer with mediastinal mass and compressive TCVO (Type 4 TCVO), presented with SVC syndrome (e.g., bilateral upper extremity and facial swelling). Baseline echocardiogram demonstrated no pericardial effusion. Blunt recanalization of the TCVO was achieved. Bilateral brachiocephalocaval 11-mm VBX stent-grafts were deployed in kissing fashion. Post deployment dilation was performed in kissing fashion. Hemodynamic change was noted and immediate echocardiogram demonstrated new pericardial fluid. A venogram was performed, and no extravasation was seen. A 10-F pericardial drain was placed, with immediate return of 500 ml of blood and normalization of hemodynamics. The patient recovered without complications, and the pericardial drain was removed on PPD 2. The patient had symptoms of pericarditis and was treated with oral colchicine, 0.6 mg once daily for 30 days.

### Patient 4

A 67-year-old female with breast cancer with chronic SVC occlusion (Type 4 TCVO) and chronic SVC syndrome (e.g., bilateral upper extremity swelling and facial swelling). Baseline limited echocardiogram demonstrated a trace pericardial effusion. Bilateral upper extremity venous access was obtained. Central venography was performed. Blunt recanalization of the TCVO was achieved from bilateral upper extremities. Balloon angioplasty was performed in kissing fashion. Bilateral brachiocephalocaval 12-mm Abre stents (Medtronic; Minneapolis, MN) were deployed in kissing fashion. Post deployment dilation was performed in kissing fashion. Limited echocardiogram demonstrated unchanged trace pericardial effusion. Patient was transferred to the recovery unit post procedure in stable condition. The patient became hypotensive and tachycardic. Limited echocardiogram was performed demonstrating a pericardial effusion. An 8-F pericardial drain was placed at bedside with immediate return of 200 ml blood. Patient was brought back to the IR procedure suite. Central venography performed without extravasation seen, although a filling small defect was seen in the left stent construct. The stents were re-lined with 13-mm Viabahn stent-grafts. The patient was weaned off of vasopressors within 24 h. The pericardial drain was removed on PPD 1.

## Discussion

The pathogenesis of TCVO can be benign or malignant with acute to chronic presentation. TCVO can be graded type 1–4 by the Central Vein Work Group classification, with type 4 involving SVC obstruction [4]. Recanalization and reconstruction for TCVO has an acceptable safety profile. In a review of stent placement for treatment of SVC syndrome in 2249 patients, the adverse event rate was 5.78% (0—53.8%) and mortality was < 1% [1]. The most frequent cause of mortality was cardiac tamponade secondary to SVC injury [1]. According to Bongers et al., a 44% mortality rate was noted if cardiac tamponade occurred during SVC stent placement [5]. A case series of 39 patients with sharp recanalization in TCVO reported similar low adverse event rates of 5% overall with no mortality [6].

The parietal pericardium envelopes the great vessels at the base of the heart, with multiple recesses and sinuses which can extend cranially in the mediastinum, as high as the level of the trachea [7]. Typically, the SVC has an intrapericardial course of approximately 3.5 cm cranial to the superior cavoatrial junction, or at approximately the level of the azygos vein or right pulmonary artery [2, 7]. Traversing pericardium during sharp recanalization or perforation, rupture or tear of the SVC at this location or perforation of the right atrium can lead to hemopericardium and cardiac tamponade.

Cardiac tamponade is a life-threatening condition with classic presenting features of obstructive shock pattern with tachycardia, elevated central venous pressure and profound hypotension secondary to low stroke volume [8, 9]. Venography may demonstrate extravasation to pericardial space; however, bleeding can be occult particularly with cardiac motion on digital subtraction angiography or due to slow flow [10]. Some authors have described immediate use of echocardiography (transesophageal or transthoracic) or CT to be able to identify pericardial effusion during TCVO reconstruction [11–13]. However, mobilizing echocardiographers to the procedural suite or transferring a patient to the CT scanner could cause clinically significant delay in care.

The preferred treatment for cardiac tamponade is image-guided pericardial drainage [5, 8]. The various technical and clinical considerations of this procedure by Interventional Radiology have been described in detail previously [14].

In situations where there is suspected ongoing bleeding, surgical management with thoracotomy and repair of the defect with or without pericardial window [5, 8]. Historically, an anatomic landmark approach was used for pericardial drain placement. However, with availability of US/echocardiography and fluoroscopy, it is standard to use image guidance for placement [9]. There are no absolute contraindications for pericardiocentesis or pericardial drain placement. Options for percutaneous access includes apical (1–2 cm lateral to apex of heart in the 5th-7th intercostal space), subcostal/subxiphoid (caudal to the xiphoid and the left costal margin) and parasternal (5th left intercostal space at the sternal margin). The site should be selected based on the best view of the effusion while avoiding critical structures (e.g., lung, bowel, internal mammary and intercostal arteries). With Seldinger technique, a 16- to 18-gauge, 9 to 15 cm long needle is advanced into the pericardial effusion with sonographic guidance. A soft-tip guidewire is placed under fluoroscopic guidance and the needle is removed. Then, dilators are used to prepare the track for drain placement under fluoroscopic guidance, typically starting with 6- or 8-F dilators and up to the drain size. Finally, the drain is placed. Immediate aspiration drainage should be performed. Gentle contrast injection can confirm pericardial space, if necessary. A gravity or accordion drainage bag can be connected. After initial pericardial drain placement, constant aspiration should be performed to prevent reaccumulation within the pericardial space and return of tamponade physiology. Drainage should decrease substantially, either due to flow diversion and/or further sealing of the initial injury. Ongoing drain output may be indicative of underlying injury that may necessitate stent-graft placement or surgical exploration and repair. With the aforementioned technique for rapid detection and treatment of cardiac tamponade, the time to life-saving drainage may be substantially decreased with minimal additional procedural cost or set-up time. This technique has been employed with technical and clinical success without complication from drain placement [3].

## Conclusion

Acute hemopericardium with cardiac tamponade is rare but life-threatening adverse event of TCVO recanalization and reconstruction. The described preparatory technique ensures that interventional radiologists are equipped to perform life-saving pericardial drainage procedure rapidly and safely.

## Data Availability

Data for this study available upon reasonable request.

## References

[1] Aung EY, Khan M, Williams N, Raja U, Hamady M. Endovascular Stenting in Superior Vena Cava Syndrome: A Systematic Review and Meta-analysis. Cardiovasc Intervent Radiol. 2022;45(9):1236–54. 35821122 10.1007/s00270-022-03178-z PMC9458578

[2] O’Horo SK, Soares GM, Dubel GJ. Acute Pericardial Effusion during Endovascular Intervention for Superior Vena Cava Syndrome: Case Series and Review. Semin Intervent Radiol. 2007;24(1):82–6. 21326743 10.1055/s-2007-971198 PMC3036355

[3] Shin DS, Jackson TR, Bertino FJ, et al. Kissing Viabahn VBX stent graft reconstruction of thoracic central veins for management of superior vena cava syndrome. J Vasc Surg Venous Lymphat Disord. 2022;10(6):1279–1287.e1. 35843595 10.1016/j.jvsv.2022.05.008

[4] Dolmatch BL, Gurley JC, Baskin KM, et al. Society of Interventional Radiology Reporting Standards for Thoracic Central Vein Obstruction: Endorsed by the American Society of Diagnostic and Interventional Nephrology (ASDIN), British Society of Interventional Radiology (BSIR), Canadian Interventional Radiology Association (CIRA), Heart Rhythm Society (HRS), Indian Society of Vascular and Interventional Radiology (ISVIR), Vascular Access Society of the Americas (VASA), and Vascular Access Society of Britain and Ireland (VASBI). J Vasc Access. 2019;20(2):114–22. 30101672 10.1177/1129729818791409

[5] Bongers KS, Patel V, Gualano SK, Schildhouse RJ. Cardiac tamponade after superior vena cava stenting. BMJ Case Rep. 2020;13(6):e234345. Published 2020 Jun 29. 10.1136/bcr-2020-234345 PMC7326245 32601136

[6] Cohen EI, Beck C, Garcia J, et al. Success Rate and Complications of Sharp Recanalization for Treatment of Central Venous Occlusions. Cardiovasc Intervent Radiol. 2018;41(1):73–9. 28879566 10.1007/s00270-017-1787-x

[7] Broderick LS, Brooks GN, Kuhlman JE. Anatomic pitfalls of the heart and pericardium. Radiographics. 2005;25(2):441–53. 15798062 10.1148/rg.252045075

[8] Chiabrando JG, Bonaventura A, Vecchié A, et al. Management of Acute and Recurrent Pericarditis: JACC State-of-the-Art Review. J Am Coll Cardiol. 2020;75(1):76–92. 31918837 10.1016/j.jacc.2019.11.021

[9] Chiara De Carlini C, Maggiolini S. Pericardiocentesis in cardiac temponade: indications and practical aspects. Eur Soc Card e-Journal of Card Prac. 2017;15(19). Accessed 12 Nov 2023. https://www.escardio.org/Journals/E-Journal-of-Cardiology-Practice/Volume-15/Pericardiocentesis-in-cardiac-tamponade-indications-and-practical-aspects.

[10] Stevens DC, Butty S, Johnson MS. Superior Vena Cava Rupture and Cardiac Tamponade Complicating the Endovascular Treatment of Malignant Superior Vena Cava Syndrome: A Case Report and Literature Review. Semin Intervent Radiol. 2015;32(4):439–44. 26622107 10.1055/s-0035-1564795 PMC4640919

[11] Brant J, Peebles C, Kalra P, Odurny A. Hemopericardium after superior vena cava stenting for malignant SVC obstruction: the importance of contrast-enhanced CT in the assessment of postprocedural collapse. Cardiovasc Intervent Radiol. 2001;24(5):353–5. 11815846 10.1007/s002700001795

[12] Anand V, Maybody M, Fischer GW, Dabo-Trubelja A. Acute Hemodynamic Compromise following Superior Vena Cava Stent Placement: A Case Report. SN Compr Clin Med. 2020;2(12):2953–6. 33458570 10.1007/s42399-020-00629-x PMC7810200

[13] Da Ines D, Chabrot P, Motreff P, et al. Cardiac tamponade after malignant superior vena cava stenting: Two case reports and brief review of the literature. Acta Radiol. 2010;51(3):256–9. 20201637 10.3109/02841850903578807

[14] Herren JL, Chan H, Ray CE Jr. Pericardial Drain Placement in Interventional Radiology: An Overview. Semin Intervent Radiol. 2022;39(3):271–274. Published 2022 Aug 31. 10.1055/s-0042-1753523 PMC9433152 36062234

